# NSUN2 modified by SUMO-2/3 promotes gastric cancer progression and regulates mRNA m5C methylation

**DOI:** 10.1038/s41419-021-04127-3

**Published:** 2021-09-09

**Authors:** Yuanbo Hu, Chenbin Chen, Xinya Tong, Sian Chen, Xianjing Hu, Bujian Pan, Xiangwei Sun, Zhiyuan Chen, Xinyu Shi, Yingying Hu, Xian Shen, Xiangyang Xue, Mingdong Lu

**Affiliations:** 1 Department of Gastrointestinal Surgery, The Second Affiliated Hospital and Yuying Children’s Hospital of Wenzhou Medical University, Wenzhou, China; 2 Department of Microbiology and Immunology, School of Basic Medical Sciences, Wenzhou Collaborative Innovation Center of Gastrointestinal Cancer in Basic Research and Precision Medicine, Wenzhou Key Laboratory of Cancer-related Pathogens and Immunity, Wenzhou Medical University, Wenzhou, China; 3 Department of Obstetrics and Gynecology, The Second Affiliated Hospital and Yuying Children’s Hospital of Wenzhou Medical University, Wenzhou, China

**Keywords:** Gastric cancer, Post-translational modifications

## Abstract

The 5-methylcytosine (m5C) RNA methyltransferase NSUN2 is involved in the regulation of cell proliferation and metastasis formation and is upregulated in multiple cancers. However, the biological significance of NSUN2 in gastric cancer (GC) and the modification of NSUN2 itself have not been fully investigated. Here, we analyzed the expression level of NSUN2 in tissue microarrays containing 403 GC tissues by immunohistochemistry. NSUN2 was upregulated in GC, and that it was a predictor of poor prognosis. NSUN2 promotes the proliferation, migration, and invasion of GC cells in vitro. We also demonstrated that small ubiquitin-like modifier (SUMO)-2/3 interacts directly with NSUN2 by stabilizing it and mediating its nuclear transport. This facilitates the carcinogenic activity of NSUN2. Furthermore, m5C bisulfite sequencing (Bis-seq) in NSUN2-deficient GC cells showed that m5C-methylated genes are involved in multiple cancer-related signaling pathways. PIK3R1 and PCYT1A may be the target genes that participate in GC progression. Our findings revealed a novel mechanism by which NSUN2 functions in GC progression. This may provide new treatment options for GC patients.

## Introduction

Human gastric cancer (GC) is the fifth most common cancer worldwide and the third leading cause of cancer-related mortality [1]. Despite significant clinical and surgical improvements, the 5-year survival rate of GC is low because more than 80% of patients are diagnosed at an advanced stage [2]. Currently, surgical resection remains the most effective treatment for early GC. Chemotherapy, radiotherapy, and other new therapies, such as immunotherapy and molecular targeted therapy, also play a significant role in the prognosis [3]. However, the prognosis of metastatic, recurrent, and advanced GC is still not satisfactory, and the mechanism of GC progression and metastasis formation is poorly understood. Hence, there is an urgent need to investigate the mechanisms of GC progression to develop new therapeutic strategies.

RNA modifications, such as N6-methyladenosine (m6A), play a nonnegligible role in epigenetic gene regulation and cellular functions [4, 5], which are closely associated with numerous human diseases, including cancer [6], neurological disorders [7], and immune dysregulation [8]. Recently, 5-methylcytosine (m5C), another important RNA modification, has attracted increasing attention. Like m6A, m5C has its own methyltransferases (writers), demethylases (erasers), and binding proteins (readers). The NOP2/Sun-domain family members 1–7 (NSUN1–7), as well as the DNA methyltransferase (DNMT) homolog DNMT2, act as m5C writers in mammals and catalyze methylation at the C5 position of RNAs [9, 10]. In contrast, ten–eleven translocation 2 (TET2) oxidizes m5C into 5-hmC and then removes the methyl group [11, 12]. Subsequently, the Aly/REF export factor (ALYREF) and Y-box binding protein 1 (YBX1), characterized as readers, recognize and bind the m5C motif, and exert further biological functions [13, 14]. In addition, these regulators, in coordination with m5C modifications, are known to participate in the progression of multiple tumors. However, the expression and function of these regulators in GC are unclear.

NSUN2, also known as Myc-induced SUN-domain-containing protein (Misu), is a nucleolar RNA methyltransferase that catalyzes 5‐methylcytosine formation in RNAs, especially in messenger RNAs [13, 15]. Accumulating evidence has revealed that NSUN2 plays a nonnegligible role in a variety of biological functions, such as cellular differentiation [16], cellular proliferation [17], cellular migration [18], and participation in tumorigenesis in an m5C-dependent manner. Immunohistochemical analysis has confirmed that NSUN2 is highly expressed in various tumors, including those of the esophagus, stomach, liver, pancreas, cervix, prostate, kidney, bladder, thyroid, and breast [19]. Although NSUN2 is the most important component of the m5C methyltransferase complex, its regulatory mechanisms and distribution in human GC remain largely unknown. Furthermore, few studies have focused on NSUN2 modifications and partner proteins.

In the present study, we utilized The Cancer Genome Atlas (TCGA) data and determined the expression levels of m5C-related regulators in GC. We discovered a close association between the expression levels of these regulators and patient survival. We also demonstrated that NSUN2 is highly expressed in GC, is associated with a poor prognosis in GC patients, and promotes the proliferation and metastasis of GC cells in vitro. In addition, we observed that SUMO-2/3 closely interacts with NSUN2 and identified a SUMO-interaction motif (SIM) in NSUN2. Moreover, SUMO-2/3 stabilized NSUN2 and mediated its transport into the nucleus. We also used bisulfite sequencing to investigate m5C methylation distribution in GC mRNA. Genes with m5C nucleotides are mainly involved in multi-cancer-associated signaling pathways. Such genes include PIK3R1 and PCYT1A, which might act as the NSUN2 target genes that participate in GC progression. Collectively, our data show that m5C RNA modification is closely involved in GC. Furthermore, the methyltransferase NSUN2 is modified by SUMO-2/3 and promotes GC progression.

## Materials and methods

### Gastric cancer dataset source and bioinformation analysis

All gene expression and clinical data used in this study were sourced from the Cancer Genome Atlas (https://portal.gdc.cancer.gov) and Gene Expression Omnibus (https://www.ncbi.nlm.nih.gov/geo/), including TCGA-STAD, ACRG/GSE62254, GSE57303, and GSE15459 cohorts. Clinical data, such as survival information, were downloaded to analyze the correlation between m5C regulators and prognosis. All data were analyzed using the R (version 4.0.1) and R Bioconductor packages.

### Patients and specimens

A total of 402 patients who underwent surgical resection for GC between January 2014 and December 2016, at the First Affiliated Hospital of Wenzhou Medical University (Wenzhou, People’s Republic of China), were included in this study. None of the patients received radiotherapy or chemotherapy before surgery. All gastric adenocarcinoma tissues and 31 paired adjacent normal tissues were formalin-fixed, paraffin-embedded, and confirmed by histopathologic analysis. Clinical pathological features, such as sex, age, tumor size, depth of invasion, lymph node metastasis, and TNM stage, were assessed. Informed consent was obtained from all patients and this study was approved by the Review Board of the First Affiliated Hospital of Wenzhou Medical University, protocol number: 2019046.

### Immunohistochemistry (IHC)

The expression level of NSUN2 in tumor and normal tissues was determined using IHC on a tissue microarray (TMA), constructed as previously described [20]. Briefly, sections were incubated with anti-NSUN2 (1:800 dilution, 20854-1-AP, Proteintech, Wuhan, China), followed by incubation with an HRP-conjugated secondary antibody, visualized with DAB (Dako, Cytomation, CA, USA), and counterstained with hematoxylin. The H score was determined based on the intensity of staining and the proportion of labeled tumor cells as previously described [21]. The formula

$$\begin{array}{lll}H - Score \,=\, \sum \left( {pi \times i} \right)\\\qquad\qquad\quad =\, \left( {\% \;of\;weak\;{\mathop{{{\rm{int}}}}} ensity\;cells \times 1} \right) + \left( {\% \,of\,\bmod erate\,{\mathop{{{\rm{int}}}}} ensity\,cells\, \times 2} \right)\\ \qquad\qquad\qquad+\, \left( {\% \,of\,strong\,{\mathop{{{\rm{int}}}}} ensity\,cells \times 3} \right)\end{array}$$

where *pi* is the percentage of positive cells, and *i* is the staining intensity.

### Cell culture and transfection

Human GC cell lines BGC-823, SGC-7901, and MGC-803 were purchased from the cell bank of the Chinese Academy of Medical Sciences (Shanghai, China). The cells were cultured in Dulbecco’s Modified Eagle’s Medium (DMEM; Gibco, Thermo Fisher Scientific) supplemented with 10% fetal bovine serum (FBS; Gibco).

The full-length sequences of NSUN2 were subcloned into a pcDNA3.1 (+) vector. The mutants of NSUN2 (C271A, C321A, and the relevant Cas9–gRNA-resistant mutants) were conducted through homologous recombination. All plasmids were validated by DNA sequencing. The small-interfering RNA (siRNA) oligonucleotides against NSUN2 and SUMO-2/3 were synthesized by RiboBio (Guangzhou, China; Supplementary Table 1). Transfection was performed using Lipofectamine 2000 reagent (Invitrogen Life Technologies®, Carlsbad, CA, USA) according to the manufacturer’s instructions.

### Generation of NSUN2-knockout GC cell lines and rescue assays

NSUN2-knockout GC cell lines were constructed using the CRISPR–Cas9 gene-editing system. Lentiviruses containing Cas9-guide RNA targeting sequences (5′-TGTTCTCCTTGACGATCTCG-3′) were designed and synthesized by HANBIO (Shanghai, China). Lentivirus infection was performed on GC cells at 80% confluency, with a multiplicity of infection (MOI) of 80. The cells were selected after culture for one week in a medium containing 2 μg/ml puromycin (MCE, Shanghai, China). Monocolonies were picked, and the knockout efficiency was determined by western blotting.

### Immunofluorescence

After transfection, the cells were washed with PBS and fixed in 4% paraformaldehyde for 15 min. Cell membranes were then permeabilized with 0.1% Triton X-100–PBS for 10 min. After blocking with 5% goat serum for 30 min at 37 °C, the cells were incubated with primary anti-HA-Tag (1:200, Cell Signaling Technology (CST), Danvers, MA, USA; #2367) and anti-SUMO-2/3 (1:200, CST; #4971) antibodies at 4 °C overnight and then incubated with DyLight 488 and DyLight 594 goat anti-rabbit/mouse secondary antibodies for 1 h at 37 °C. Afterward, the cells were incubated with 4,6-diamidino-2-phenylindole (DAPI) for 5 min. All images were captured using a fluorescence microscope (Leica, London, UK).

### Co-immunoprecipitation

For Co-IP assays, cells transfected with HA-tagged NSUN2 were lysed with NP-40 containing protease-inhibitor cocktail (MCE) and N-Ethylmaleimide (Sigma). The supernatant was collected and incubated with anti-HA magnetic beads (Thermo Fisher Scientific Inc.) for at least 2 h at 20 °C. The immunoprecipitates were then boiled in 2× SDS loading buffer and analyzed by SDS-PAGE.

### Extraction of cytoplasmic and nuclear lysates

Extraction of cytoplasmic and nuclear lysates was performed to assess the localization of NSUN2 using an Ambion PARISTM kit (Thermo Fisher Scientific Inc.) as previously described [22]. Briefly, cells were lysed on ice using a cold cell fractionation buffer for 10 min, and centrifuged at 500 × *g* for 5 min to obtain the cytoplasmic fraction. The precipitate was then lysed using a cell-disruption buffer to obtain the nuclear protein.

### Western blotting

Cells were harvested and dissolved in RIPA lysis buffer and a protease-inhibitor cocktail. Whole-cell lysates were subjected to SDS-PAGE and transferred to PVDF membranes (Bio-Rad, Hercules, CA, USA). After blocking and incubating with specific primary and secondary antibodies, the proteins on the membranes were visualized using the Bio-Rad ChemiDoc^®^ Touch Imaging System (Bio-Rad).

The antibodies used included HA-Tag (1:1000, CST), NSUN2 (1:5000, Proteintech), SUMO-2/3 (1:1000, CST, #4971), GAPDH (1:1000, Proteintech; 60004-1-Ig), α-Tubulin (1:1000, CST; #3873), and Lamin A/C (1:1000, CST; #4777).

### RNA isolation and quantitative reverse-transcription polymerase chain reaction (qRT-PCR)

Total RNA was extracted from the GC cell lines using TRIzol reagent (Invitrogen; Thermo Fisher Scientific, Carlsbad, CA, USA), and reverse-transcribed using a ReverTraAce^®^ qPCR RT Kit (Toyobo, Tokyo, Japan) according to the manufacturer’s instructions. The complementary DNA was then amplified to detect SUMO-2/3, PIK3R1, and PCYT1A by qRT-PCR using a SYBR Green Master mix (QIAGEN) according to the manufacturer’s protocol. All tests were repeated independently three times. The mRNA expression was normalized to the expression of GAPDH mRNA and calculated using the 2-ΔΔCt method. The PCR primers used are listed in Supplementary Table 2.

### Cell proliferation and colony-formation assays

Cell proliferation was assessed using Counting Kit-8 (CCK-8; Dojindo, Kumamoto, Japan). Briefly, cells were seeded into 96 well plates (5000 cells/well, five replicates). Then, cells were transfected and treated with CCK-8 regent. The OD value at 450 nm was measured using a microplate reader. For colony formation assays, transfected cells were placed in six well plates (200 cells/well) for two weeks. Cells were then fixed with 4% paraformaldehyde for 10 min and stained with 0.1% crystal violet for 10 min. The number of colonies was counted.

### 5-Ethynyl-2′-deoxyuridine (EdU) assay

The effect of NSUN2 on GC cell proliferation was also tested using an EdU assay kit (Cell Light EdU DNA imaging Kit, RiboBio). Briefly, 10,000 cells per well were added to 96-well plates (three replicates). After transfection, EdU (50 μM) was added and incubated at 37 °C for 2 h. Subsequently, the cells were washed with PBS and fixed with 4% paraformaldehyde for 30 min. Next, 0.1% Triton X-100–PBS was used for cell membrane permeabilization for 10 min. After washing with PBS three times, cells were stained with EdU Apollo^®^567 for 30 min, and DNA was stained with Hoechst 33342 for 20 min. The proportion of EdU-positive cells was visualized by fluorescence microscopy.

### Transwell migration and invasion assays

Transwell migration and invasion assays were performed as described previously [22]. For the invasion assay, the chambers were coated in advance with Matrigel (BD Pharmingen, San Jose, CA, USA). Then 1 × 105 transfected cells were seeded into the upper chamber in serum-free medium, and medium with 10% FBS was added to the basolateral chamber. After 24-h incubation for the migration assay and 36-h incubation for the invasion assay, cells were fixed with 4% paraformaldehyde for 10 min and stained with 0.1% crystal violet for 10 min. The cells were photographed in five randomly selected visual fields under a microscope (Leica, London, UK).

### RNA-Bis-Seq and bioinformatics analyses

RNA Bis-Seq and bioinformatics analyses were performed as previously described [23]. Briefly, rRNA-depleted RNA was bisulfite-converted and purified. Then, RNA libraries were constructed and sequencing was performed on an Illumina Novaseq instrument with 150-bp paired-end reads. After 3′-adaptor trimming, low-quality reads were removed using cutadapt software. Clean reads of Bis-treated libraries were then mapped to UCSC HG19 using meRanGs software. The m5C sites within the genome were extracted by meRanCall. Differentially methylated sites (DMSs) were identified using meRanCompare. Only sites with coverage depth (methylated C number + nonmethylated C number) ≥10, m5C methylation level ≥0.1, and methylated cytosine depth ≥5 were considered as credible m5C sites. The m5C sites were annotated using BEDTools intersectBed and the distribution of m5C sites was plotted using MetaPlotR software. The GO and KEGG pathways of differentially methylated genes (DMGs) were analyzed by Metascape.

### Statistical analysis

All statistical analysis was performed using SPSS 22.0 software (SPSS Inc., Chicago, IL, USA), GraphPad Prism software (version 7.0), and R (https://www.r-project.org/, version 3.6.2). Continuous variables were expressed as the mean ± standard deviation (SD) and were analyzed by unpaired Student’s t tests for the comparison of two groups when the variances are equal. For survival analysis, Kaplan–Meier method was used and a log-rank test was adopted for comparison. *P < 0.05, **P < 0.01, and ***P < 0.001 were considered statistically significant.

## Results

### Expression landscape and clinical correlation of m5C regulators in GC

**Fig. 1:**
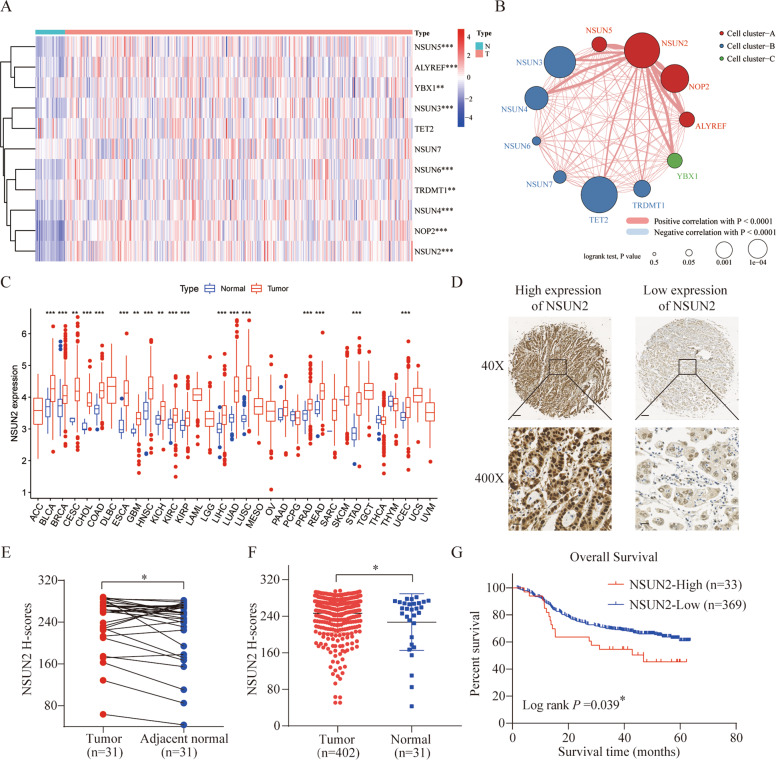
Expression landscape and clinical correlation of RNA m5C regulators in gastric cancer (GC) and NSUN2. A A heatmap was used to visualize the expression of m5C regulators in TCGA-STAD database. Red and blue regions represent higher and lower expression levels, respectively. **B** Interactions between m5C regulators in GC. The size of each circle represents the influence of each regulator on prognosis, as determined via the log-rank test. The lines connecting regulators represent their interactions, and the thickness of the line represents the correlation strength. A positive correlation is marked with blue and a negative correlation with red. The regulator clusters A–C were marked red, blue, and green, respectively. **C** Expression of NSUN2 across 33 different cancers in TCGA database. **D** Representative images of immunohistochemical staining for NSUN2 protein in a tissue microarray (×40: scale bar = 100 μm; ×400: scale bar = 10 μm) and (**E** and **F**) the differential expression of NSUN2 between tumor and adjacent normal tissue. **G** Kaplan–Meier OS analysis of NSUN2 expression in GC patients. *P < 0.05, **P < 0.01, and ***P < 0.001.

To evaluate the expression profile of m5C regulators in GC, we extracted m5C-related gene sequencing data from TCGA Stomach Adenocarcinoma (STAD) projects. The overall expression level is shown in a heatmap (Fig. 1A). The m5C regulators were generally highly expressed in GC samples compared to that of control samples. Additionally, the m5C regulator network depicted a landscape of their interactions and effects on the overall survival of patients with GC (Fig. 1B). The results showed that NSUN2 was closely connected with other m5C regulators and the global expression level of m5C regulators was significantly correlated with the overall survival of patients.

### NSUN2 was highly expressed in GC and associated with a poor prognosis

According to TCGA data, we found that NSUN2 was overexpressed in various cancers (Fig. 1C); thus, we speculated that NSUN2 might act as a common oncogene that participates in human cancer progression. To investigate the patterns of expression of NSUN2 and the clinical implication for GC patients, we performed IHC staining for NSUN2 using our archived GC tissue microarray. IHC showed that NSUN2 was expressed mainly in the nucleus of cancer cells, and partially expressed in the cytoplasm (Fig. 1D). NSUN2 was more highly expressed in tumor tissues than in adjacent normal tissues (Fig. 1E and F). Based on the different H scores, obtained by IHC, we divided these patients into NSUN2 high expression (*n* = 33) and low expression (*n* = 369). No significant correlations were observed between NSUN2 and age, T stage, N stage, tumor size, or differentiation (*P* > 0.05) (Table 1). Kaplan–Meier survival analysis showed that GC patients with high NSUN2 expression had a lower overall survival (OS) than those with low NSUN2 expression (hazard ratio, 1.93; 95% confidence interval, 1.03–3.62; log-rank *P* = 0.039) (Fig. 1G). In the univariate analysis of OS, the expression of NSUN2 was identified as an independent risk factor (HR, 1.576; 95% CI, 1.025–2.424; *P* = 0.038). However, multivariate analysis showed that high expression of NSUN2 was not an independent predictor of OS (HR, 1.55; 95% CI, 0.93–2.59, *P* = 0.09) (Table 2). Overall, these results demonstrate that NSUN2 is highly expressed in GC tissues and is associated with a poor prognosis in GC patients.

**Table 1:** Clinicopathological features and NSUN2 in GC.

Variables	All patients (*n* = 402)	Low (*n* = 369)	High (*n* = 33)	*P* value
*Gender*				0.010^*^
Female	95	93	2	
Male	307	276	31	
*Age*				0.522
<65	192	178	14	
≥65	210	191	19	
*T stage*				0.621
T1 + T2	125	116	9	
T3 + T4	277	253	24	
*N stage*				0.570
N0 + N1	238	220	18	
N2 + N3	164	149	15	
*Tumor size*				0.643
<4.5 cm	253	231	22	
≥4.5 cm	149	138	11	
*Stage*				0.522
I + II	192	178	14	
III + IV	210	191	19	
*Differentiation*				
Moderate + Well	124	113	11	0.747
Poor	278	256	22	

*Statistically significant (*P* < 0.05).

**Table 2:** Univariate and multivariate Cox regression analysis of overall survival in patients with gastric cancer.

Variables	Univariate cox analysis		Multivariate cox analysis	
	HR (95% CI)	*P* value	HR (95% CI)	*P* value
Gender (male vs. female)	1.45(0.95–2.20)	0.082	1.56 (1.02–2.39)	0.042*
Age (≥65 vs. <65)	1.86(1.33–2.61)	<0.001*	1.84 (1.31–2.58)	<0.001*
T (T3 + T4 vs. Tis + T1 + T2)	3.87(2.39–6.27)	<0.001*	1.78 (0.95–3.34)	0.072
N (N2 + N3 vs. N0 + N1)	2.28(1.64–3.16)	<0.001*	1.13 (0.74–1.74)	0.557
Stage (III + IV vs. I + II)	3.15(2.19–4.55)	<0.001*	1.86(1.16–2.97)	0.01*
Differentiation (Poor vs. Moderate + Well)	2.10(1.40–3.13)	<0.001*	1.71(1.13–2.69)	0.01*
Tumor size (>4.75 cm vs. ≤4.75 cm)	2.20 (1.59–3.05)	<0.001*	1.67(1.19–2.34)	0.03*
NSUN2 expression (high vs. low)	1.69(1.02–2.81)	0.042^*^	1.55(0.93–2.59)	0.09

^*^Statistically significant (*P* < 0.05).

### NSUN2 promotes cell proliferation and metastasis of GC cells

**Fig. 2:**
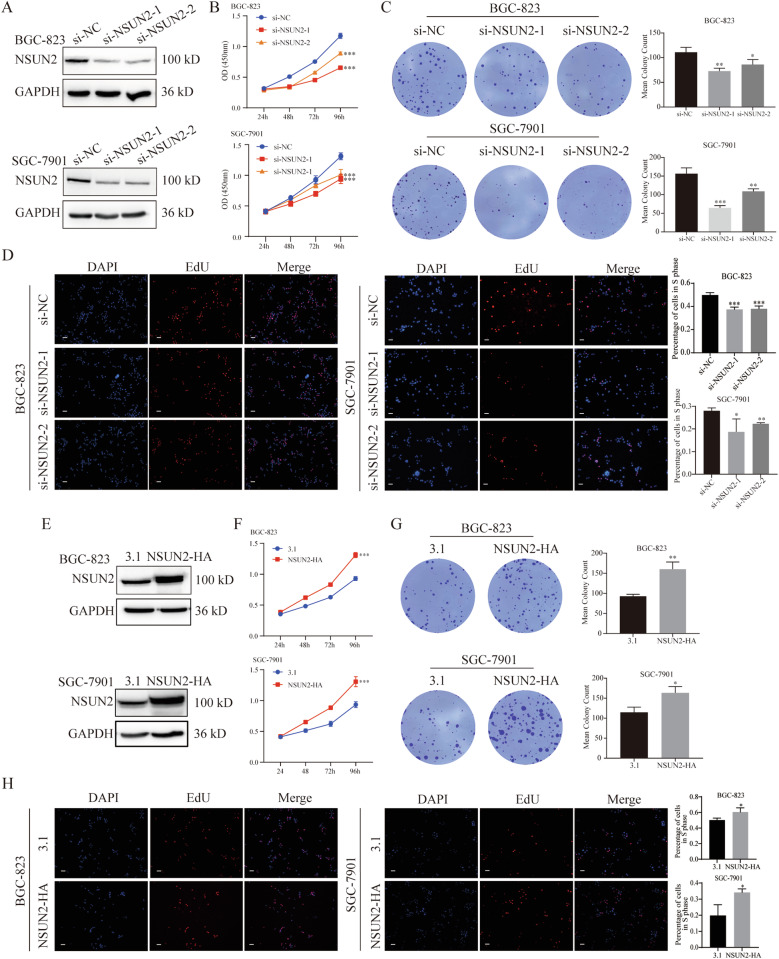
NSUN2 promotes the growth of human gastric cancer (GC) cells. (**A** and **E**) Western blot analysis of NSUN2 knockdown and overexpression efficiency in BGC-823 and SGC-7901 cells. (**B**–**D** and **F**–**H**) The proliferation of cells under NSUN*2* knockdown and overexpression was determined via CCK-8, colony formation, and EdU assays. Scale bar, 100 μm, *P < 0.05, **P < 0.01, and ***P < 0.001.

To investigate the tumorigenic role of NSUN2 in GC cells, we knocked down its expression with siRNAs in BGC-823 and SGC-7901 cells. NSUN2 expression was significantly decreased, as determined by western blotting (Fig. 2A). To determine the regulatory effect of NSUN2 on the proliferation of GC cells, we performed CCK-8 and 5-ethynyl-2′-deoxyuridine (EdU) assays. Interestingly, downregulation of NSUN2 led to a decreased cell proliferation rate in both BGC-823 and SGC-7901 cells (Fig. 2B and D). In contrast, NSUN2 overexpression resulted in a marked increase in the cell proliferation rate compared to that in the control group (Fig. 2E–I). Colony-formation assays were performed to determine the long-term impact of NUSN2 on GC cell proliferation. We found that fewer colonies formed in the NSUN2-knockdown group after two weeks, whereas there was an increase in the number of colonies formed in the NSUN2-overexpression group compared with the control group (Fig. 2C and H). Overall, these results showed that NSUN2 promotes the proliferation of GC cells.

**Fig. 3:**
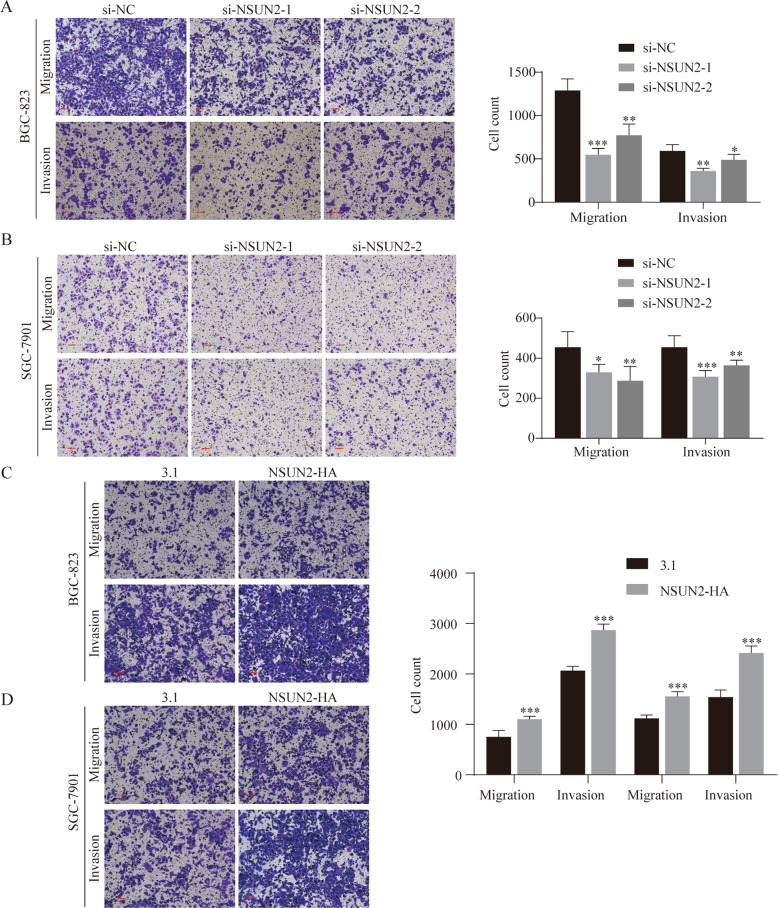
NSUN2 promotes the migration and invasion of gastric cancer (GC) cells. **A**–**D** Representative images of Transwell assays with BGC-823 and SGC-7901 cells under NSUN2 knockdown. The numbers of migrating and invading cells are presented in the right panel. Scale bar, 100 μm. *P < 0.05, **P < 0.01, and ***P < 0.001.

To further explore the role of NSUN2 in GC progression, we performed migration and invasion assays with NSUN2 knockdown and overexpression in BGC-823 and SGC-7901 cells. As shown in Figs. 3A and 3B, the number of migrating and invading cells significantly decreased in GC cells transfected with si-NSUN2 compared with si-NC. In contrast, there was an increase in the migration and invasion abilities of GC cells overexpressing NSUN2 compared with that of control cells (Fig. 3C, D). In addition, we also performed transwell assays with other types of cancer cells, such as breast cancer (Hs 578t), hepatocellular carcinoma (PLC/PRF/5), thyroid cancer (FTC-133), and esophageal cancer (KYSE-150), transfected with si-NSUN2, which contributed inconsistent results (Fig. S1). Collectively, these results indicate that NSUN2 levels are closely related to the migration and invasion properties of GC cells; hence, NSUN2 may act as a pan-cancer oncogene.

### SUMO-2/3 modulates NSUN2’s protein stability and subcellular localization

**Fig. 4:**
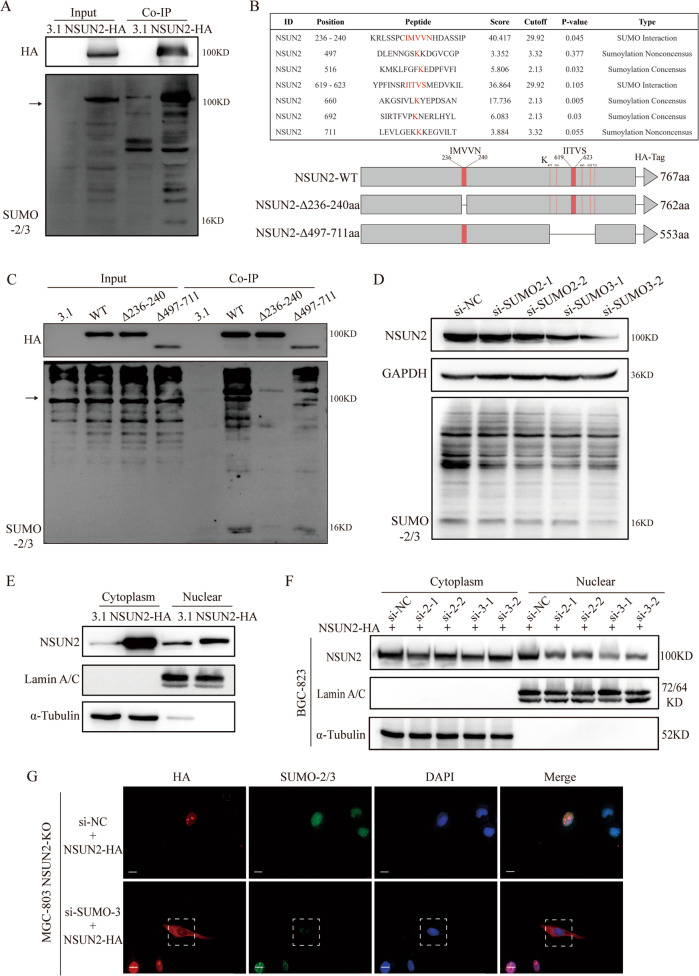
SUMO-2/3 interacts with NSUN2 and maintains its stability and facilitates its nuclear translocation. **A** SUMO-2/3 directly interacted with NSUN2, as determined via Co-IP. **B** Schematic representation of the SUMOylation sites and SIMs in NSUN2 predicted via GPS-SUMO (http://sumosp.biocuckoo.org/online.php). The schematic below represents the mutant plasmids harboring NSUN2 wildtype, NSUN2–Δ236–240aa, and NSUN2–Δ497–711aa. **C** Co-IP assays showed that SUMO-2/3 interacted with NSUN2 in SIM (236–240aa). **D** The expression level of NSUN2 and SUMO-2/3 after SUMO-2/3 knockdown. **E** Expression of NSUN2 in the cytoplasm and nucleus after NSUN2-HA transfection. **F** Expression of NSUN2 in the cytoplasm and nucleus in BGC-823 cells transfected with NSUN2-HA and si-SUMO-2/3. **G** Representative immunofluorescence images show that overexpressed NSUN2 significantly accumulates in the cytoplasm and that NSUN2 nuclear transport was blocked in NSUN2-knockout SGC-823 cells transfected with NSUN2-HA and si-SUMO-2/3. The white rectangle indicates the cells with SUMO-2/3 knockdown. Scale bar, 20 μm.

To explore the molecular mechanism by which NSUN2 promotes GC progression, we attempted to identify the proteins that interact with NSUN2 by performing co-immunoprecipitation (co-IP) and mass spectrometry analyses (Fig. S2A). Among the identified NSUN2-interacting proteins, SUMO-2/3 attracted our attention because SUMOylation is an important post-translational protein modification that is widespread in eukaryotes [24]. Correlation analysis showed a positive correlation between NSUN2 and SUMO-2/3 (Fig. S2B). Furthermore, the co-IP results showed that SUMO-2/3 was pulled down with NSUN2-HA, demonstrating a direct interaction (Fig. 4A). During our identification of the specific SUMO-conjugation site of NSUN2, we found two SUMO-interaction motifs (SIMs) and five SUMOylation consensus sites using a GPS-SUMO tool (Fig. 4B). Next, we constructed two mutant plasmids that truncated 236–240aa and 497–711aa of NSUN2. We transfected fulllength, Δ236–240aa, and Δ497–711aa of NSUN2-HA into BGC-823 cells and performed Co-IP. As shown in Fig. 4C, the interaction between NSUN2 and SUMO-2/3 almost disappeared after NSUN2–Δ236–240aa transfection, while there was a slight decrease in interaction after NSUN2–Δ497–711aa transfection. These results demonstrate that the interaction between NSUN2 and SUMO-2/3 mainly depends on the SIM (236–240aa) of NSUN2.

SUMOylation can influence protein stability and localization [25]. Therefore, we attempted to explore whether NSUN2 SUMOylation affected its protein level. First, we transfected si-RNAs against SUMO-2/3 into BGC-823 cells and measured NSUN2 protein levels by western blotting. As shown in Fig. 4D and S2C–D, the total protein level of NSUN2 was little decreased after SUMO-2/3 knockdown. In order to investigate whether SUMOylation could alter NSUN2 subcellular localization, we transfected NSUN2-HA plasmid and found that NSUN2 overexpression resulted in significantly increased NSUN2 expression in the cytoplasm and the nucleus (Fig. 4E and S2E). Next, we cotransfected NSUN2-HA and SUMO-2/3 si-RNAs into cells and isolated cytoplasmic and nuclear proteins. Intriguingly, the level of NSUN2 protein had a little decrease in the cytoplasm, while it decreased significantly in the nucleus after SUMO-2/3 knockdown (Fig. 4F and S2F). Immunofluorescence (IF) assays were performed in NSUN2-knockout MGC-803 (Fig. 4G) and BGC-823 (Fig. S2G) cells to determine the subcellular distribution of NSUN2 after NSUN2-HA and si-SUMO-3-2 transfection. The results further demonstrated that SUMO-2/3 knockdown inhibited NSUN2 translocation to the nucleus. Subsequently, the SUMO-interaction region-deficient NSUN2 plasmids were transfected into the NSUN2-knockout MGC-803 cells and the localization of NSUN2 was determined by IF assays. Importantly, NSUN2–Δ236–240aa was mostly located in the cytoplasm, whereas NSUN2-WT and NSUN2–Δ497–711 were mainly expressed in the nucleus with some expression detected in the cytoplasm (Fig. S2H). These results indicate that SUMO-2/3 plays a vital role in the nuclear transportation of NSUN2, and further confirm the exact region of interaction between NSUN2 and SUMO-2/3. Moreover, the Transwell assays performed with NSUN2-knockout BGC-823 cells further demonstrated that NSUN2 overexpression significantly promotes migration, whereas the simultaneous silencing of SUMO-2/3 partially inhibits the oncogenic effect of NSUN2 overexpression (Fig. S2I). Taken together, these results imply that the SUMOylation of NSUN2 influences its stability and mediates its importation into the nucleus, where it exerts further biological and carcinogenic effects.

### NSUN2 promotes gastric progression via both m5C-dependent and -independent mechanisms

**Fig. 5:**
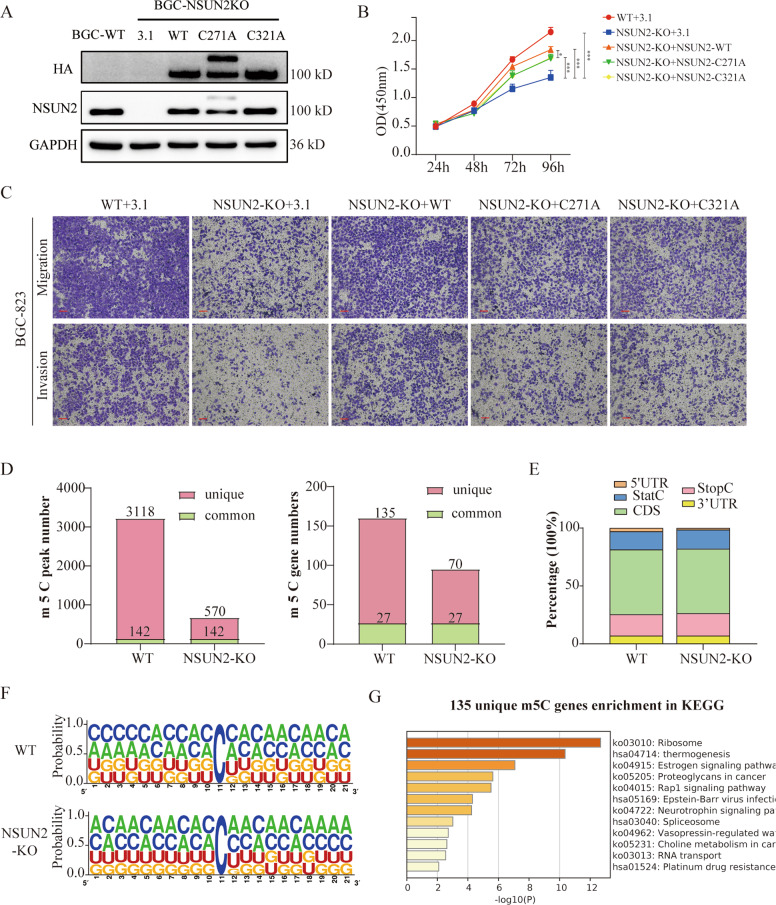
Oncogenicity of NSUN2 partly relies on m5C modification. **A** The C271A and C321A mutant constructs of NSUN2 were detected in NSUN2-knockout BGC-823 cells via western blot. **B** and **C** The proliferation and migration-rescue assays of NSUN2 as well as its mutants’ overexpression in NSUN2 knockout BGC-823 cells. Scale bar, 100 μm. **D** Bis-Seq determined the m5C peak numbers and m5C genes in WT and NSUN2-knockout BGC cells. **E** Distribution of mRNA m5C sites in GC. **F** m5C sequence frequency logo in GC transcripts. **G** Bar graph of enriched KEGG terms across unique m5C-modified genes in the WT group. Scale bar, 10 μm. *P < 0.05, **P < 0.01, and ***P < 0.001.

To determine whether the oncogenic function of NSUN2 depends on its m5C methyltransferase activity, we generated two enzymatic dead mutants of NSUN2 by introducing point mutations in releasing (cysteine 271) and catalytic (cysteine 321) sites (Fig. 5A), which had the potential to totally destroy its m5C enzymatic activity [26]. By overexpressing NSUN2 wild-type and mutant plasmids in the BGC-823 NSUN2-knockout cell line, we found that both the wild-type and enzymatic dead mutants of NSUN2 were able to partly rescue the ability of proliferation and metastasis of GC cells, and the wild-type NSUN2 presented more significant functions (Fig. 5B, C and S3A). These results indicated that NSUN2 promoted gastric progression via both m5C-dependent and -independent mechanisms.

To explore m5C-dependent functions of NSUN2, we performed RNA Bis-Seq to map the transcriptome-wide m5C modifications after NSUN2 knockout in GC cells. In total, we identified 3260 and 712 m5C peaks and 135 and 70 unique genes in WT and NSUN2-knockout cells, respectively (Fig. 5D). This confirmed that the m5C modification in eukaryotic mRNAs is mainly catalyzed by NSUN2. According to the localizations of the m5C peaks in the RNA transcripts, we divided them into 5′-untranslated regions, StartC, coding sequences (CDS), StopC, and 3′-untranslated regions (Fig. 5E). Similar to previous studies [13], the m5C sites were localized in CG-rich environments (Fig. 5F). Gene ontology (GO) analysis revealed that 135 unique m5C transcripts were significantly enriched in gene sets involved in mRNA metabolism and translational initiation (Figure S3B). KEGG analysis showed that NSUN2-mediated m5C-modified genes were significantly enriched in multiple cancer-related signaling pathways, such as the Rap1 signaling pathway, platinum drug resistance, and the regulation of cell cycle processes (Fig. 5G). These data imply that the NSUN2-mediated m5C modification in GC transcripts is closely associated with RNA metabolism and cancer development.

**Fig. 6:**
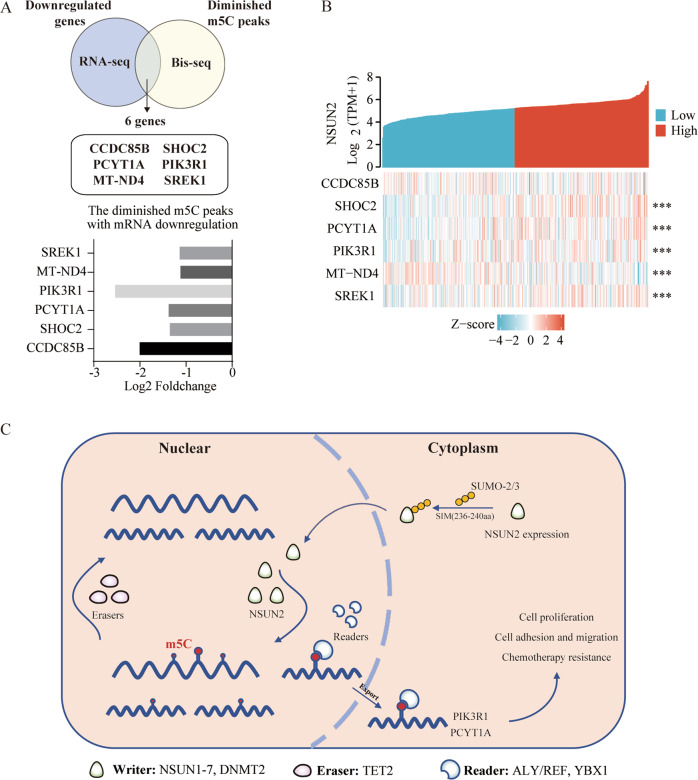
Potential m5C modification candidates of NSUN2 directly target genes. **A** Potential direct targets of NSUN2 and the change in their mRNA levels. **B** The coexpression heatmap between NSUN2 and its target genes based on the TCGA-STAD dataset. **C** Proposed model for the functional landscape of the SUMO-2/3–NSUN2–m5C modification in gastric cancer.

In addition, we attempted to investigate whether changes in m5C peaks would affect the mRNA expression level. We filtered the decreasing m5C peaks with downregulated genes in NSUN2-KO BGC-823 cells and identified six candidate targets of NSUN2, including CCDC85B, SHOC2, PCYT1A, MT-ND4, SREK1, and PIK3R1 (Fig. 6A). The coexpression heatmap showed that the expression of NSUN2 was closely related to all these genes except CCDC85B (Fig. 6B). We also found that high expression levels of PIK3R1 and PCYT1A corresponded to a poor prognosis from the TCGA-STAD dataset (Fig. S3C). Then, we confirmed that the expression levels of PIK3R1 and PCYT1A decreased significantly after NSUN2 knockout through qRT-PCR (Fig. S3D). Therefore, we speculate that PIK3R1 and PCYT1A might act as the target genes of m5C modified by NSUN2. Collectively, these results indicate that m5C modification mediated by NSUN2 participated in the progression of GC.

## Discussion

GC is one of the most life-threatening malignancies worldwide, and more than 40% of the new cases and deaths occur in China [27]. With the rapid development of early diagnostic and therapeutic strategies, especially the application of immunotherapy and targeted therapy, the overall survival rate of GC has improved [28]. Nonetheless, the prognosis of advanced GC patients remains poor. New effective molecular diagnostics and therapeutic targets are urgently needed. Here, we demonstrated that the RNA methyltransferase NSUN2 is highly expressed in GC and acts as a prognostic biomarker that correlates with poor prognosis. Downregulation of NSUN2 can inhibit GC cell proliferation and metastasis in vitro, whereas overexpression of NSUN2 can promote GC cell proliferation and metastasis. We demonstrated for the first time that SUMO-2/3 interacts with NSUN2 and mediates its importation into the nucleus, where it exerts further biological effects. We also determined the distribution of m5C in GC mRNA and further speculated that PIK3R1 and PCYT1A are the target genes of m5C and promote GC progression.

5-methylcytosine, an RNA modification that is attracting increasing attention, can dynamically regulate a variety of biological functions through its regulators. He et al. [29] analyzed the roles of m5C regulators in hepatocellular carcinoma and found that ALYREF and NSUN4 may act as tumor oncogenes, predicting a poor prognosis. Xiang et al. [30] found that nine m5C modulators were highly expressed across gastrointestinal cancers and only NSUN5 and ALYREF were significantly correlated with the overall survival of patients in STAD data. Here, we showed that the expression of m5C regulators was dysregulated in GC, which is similar to the results of previous studies, indicating that RNA m5C modification might be closely involved in the progression of GC. Furthermore, we found that 11 diverse m5C regulators, especially NSUN2, were closely connected with others and presented a significant correlation for the prognosis of GC patients.

Accumulating evidence has shown that NSUN2 plays a vital role in the pathogenesis and progression of multiple cancers. Frye et al. [31] found that NSUN2 is upregulated in tumors and is essential for Myc-induced proliferation. Gao et al. [32] found that NSUN2 is highly expressed in gallbladder carcinoma and could promote gallbladder carcinoma progression via its interacting partner, RPL6. Chen et al. [14] revealed that the NSUN2/YBX1/m5C–HDGF axis could promote the pathogenesis of bladder cancer. Sun et al. [33] demonstrated that NSUN2 mediates the lncRNA H19 m5C modification and promotes the occurrence and development of hepatocellular carcinoma. Furthermore, NSUN2 acts as an oncogene and promotes cell proliferation in GC by destabilizing p57Kip2 mRNA in an m5C-dependent manner [34]. In the present study, we found that NSUN2 was highly expressed in GC, which was correlated with a poor prognosis. Furthermore, we found that NSUN2 promoted cell proliferation in GC cells and promoted migration and invasion in various human tumor cells, including GC, breast cancer, hepatocellular carcinoma, thyroid cancer, and esophageal cancer. These results revealed that NSUN2 might act as a common oncogene during pan-cancer tumorigenesis, which provides new insights into the pathogenesis of tumor development.

NSUN2 is predominantly localized in the nucleus or the nucleus and cytoplasm, and may play a role in complex biological functions, depending on its subcellular localization [35]. NSUN2 is required for mitotic spindle stability, and its subcellular distribution changes from the nucleus to the mitotic spindle during the cell cycle [36]. As an m5C methyltransferase, NSUN2 mainly exerts its methyltransferase activity in the nucleus [31]. Current research on NSUN2 in tumorigenesis is generally aimed at understanding its function in RNA modification and further exploring its downstream target genes. However, little research has focus on the regulation of NSUN2 subcellular localization, especially its nuclear transport. Here, we first demonstrated that NSUN2 directly interacted with SUMO-2/3 and then speculated that SUMOylation of NSUN2 may influence its stability and mediate its nuclear transport.

SUMOylation is a major regulatory post-translational modification. The addition of SUMO to target proteins occurs via covalent binding to specific lysine residues and noncovalent binding to the SIMs of substrates [37, 38]. SUMOylation can alter the stability, subcellular localization, and biological activity of substrate proteins [39]. The relationship between NSUN2 and SUMOylation within the context of cancer progression and metastasis remains unclear. Herein, we confirmed that SUMO-2/3 interacted with NSUN2 and identified the 236–240aa region as the SIM of NSUN2. We found that SUMO-2/3 modified NSUN2 to maintain its stability. NSUN2-HA overexpression and SUMO-2/3 knockdown in GC cells resulted in a significant decrease of nuclear NSUN2. IF results indicated that SUMO-2/3 knockdown resulted in the significant cytoplasmic accumulation of NSUN2, presumably due to a block of its nuclear transport. Moreover, the 236–240aa-deficient NSUN2 could not enter the nucleus in contrast to wild-type and NSUN2–Δ497–711 NSUN2, which further demonstrated that SUMO-2/3 modified NSUN2 through interacting with the latter’s SIM (236–240aa) to mediate nuclear localization. To determine whether SUMOylation and nuclear localization of NSUN2 were required for its function, Transwell assays were performed. These results indicated that NSUN2 SUMOylation was indeed required for its biological effects. Nevertheless, the specific covalent SUMOylation-binding sites and their role in NSUN2 function remain unclear and will need to be fully characterized in future studies.

The majority of research agrees that as the main methyltransferase of m5C, NSUN2 functions in an m5C-dependent manner. However, in this study, we found that catalytically inactive NSUN2 enzymes partially retained their oncogenic effects, although the wild-type NSUN2 was much more potent. These observations indicated that NSUN2 promoted gastric carcinogenesis via both m5C-dependent and -independent mechanisms. Similar to our results, Hussain et al. [36] demonstrated that the methyltransferase activity of NSUN2 is dispensable in its role in regulating spindle stability and cell cycle. Nevertheless, the underlying mechanism of NSUN2 function in cancer requires further exploration.

RNA m5C dysregulation has been shown to play multiple roles in human malignancy through the modification of oncogenes. The distribution of m5C sites in mRNA is enriched in the coding sequences and 3′ UTRs, especially downstream of the translation-initiation site, which is highly conserved in mammals and is closely associated with numerous human diseases [13]. Herein, RNA-Bis-Seq was performed to determine the m5C modification distribution in GC mRNA, which was similar to the approach of previous reports. NSUN2 knockout significantly decreased m5C levels in GC cells, further demonstrating that the m5C modification was mainly catalyzed by NSUN2. GO analysis of post-NSUN2-knockout hypomethylated genes showed that these were mainly enriched in metabolic and translational initiation processes. KEGG pathway analysis indicated the enrichment of hypomethylated genes in the Rap1 signaling pathway, platinum drug resistance, and the regulation of cell cycle processes.

m5C mainly affects the stability of target mRNA. Therefore, six genes—CCDC85B, SHOC2, PIK3R1, PCYT1A, MT-ND4, and SREK1—were identified by further filtering downregulated genes with diminished m5C peaks. Feng et al. [40] reported that CCDC85B is an oncogene that promotes the proliferation and invasion of lung cancer cells by activating the AKT/GSK3β/β-catenin signaling pathway. SHOC2 is a positive regulator of the Ras pathway, regulating E-cadherin turnover and promoting cell migration [22]. Geng et al. [41] found that SHOC2 is closely associated with aggressive clinical characteristics and predicts a poor prognosis in breast cancer patients. Further, *SHOC2* knockdown inhibited the proliferation of breast cancer cells. PCYT1A, a choline-phosphate cytidylyltransferase, was reported to act as a tumor suppressor in lung adenocarcinoma [42]. PIK3R1 is the regulatory subunit of PI3K (p85α), which binds to the p110α subunit to directly activate the PI3K pathway and subsequent pathways. The PI3K pathway participates in the development of various cancers [43–45]. In addition, we found that PIK3R1 and PCYT1A were associated with a poor prognosis of GC patients in the TCGA dataset. We presumed that NSUN2 could catalyze the m5C modification of PIK3R1 and PCYT1A, and thus regulate their expression, promoting GC carcinogenesis. However, whether m5C modification affects the stability of these transcripts or the efficiency of their translation is still unknown. Additional functional rescue experiments are required to clarify the link between NSUN2-mediated m5C and genes involved in the development of GC. Further, more m5C readers have to be identified in order to explore the modification’s effects.

In summary, we demonstrated that NSUN2 is upregulated and associated with a poor prognosis in GC. NSUN2 promoted the progression and metastasis of tumor cells in vitro. Furthermore, SUMO-2/3 was shown to interact with the SIM (236–240aa) of NSUN2 through noncovalent bonds in order to mediate the latter’s nuclear transport. In addition, we determined m5C distribution in GC mRNA and showed that the m5C-modified genes were mainly involved in multiple cancer-associated signaling pathways. Therefore, the SUMOylation–NSUN2–m5C axis may represent a new diagnostic and therapeutic target for GC and pan-cancer treatment (Fig. 6C).

### Supplementary information

Supplementary Legends

Supplementary Table 1

Supplementary Table 2

Supplementary Figure 1

Supplementary Figure 2

Supplementary Figure 3
