# Correction: NSUN2 modified by SUMO-2/3 promotes gastric cancer progression and regulates mRNA m5C methylation

**DOI:** 10.1038/s41419-024-06859-4

**Published:** 2024-07-11

**Authors:** Yuanbo Hu, Chenbin Chen, Xinya Tong, Sian Chen, Xianjing Hu, Bujian Pan, Xiangwei Sun, Zhiyuan Chen, Xinyu Shi, Yingying Hu, Xian Shen, Xiangyang Xue, Mingdong Lu

**Affiliations:** 1 Department of Gastrointestinal Surgery, The Second Affiliated Hospital and Yuying Children’s Hospital of Wenzhou Medical University, Wenzhou, China; 2 Department of Microbiology and Immunology, School of Basic Medical Sciences, Wenzhou Collaborative Innovation Center of Gastrointestinal Cancer in Basic Research and Precision Medicine, Wenzhou Key Laboratory of Cancer-Related Pathogens and Immunity, Wenzhou Medical University, Wenzhou, China; 3 Department of Obstetrics and Gynecology, The Second Affiliated Hospital and Yuying Children’s Hospital of Wenzhou Medical University, Wenzhou, China

Correction to: *Cell Death and Disease* 10.1038/s41419-021-04127-3, published online 09 September 2021

There were some mistakes in Figures and Table 2:

1. Figure 2H. The BGC-823 3.1 group DAPI image was accidentally used in the SGC-7901 3.1 group DAPI.
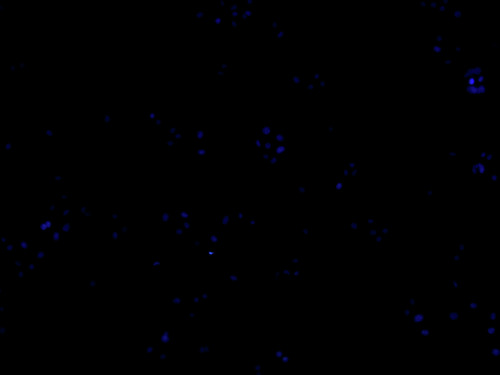


2. Supplementary Figure 1. Fig S1C KYSE-150 si-NSUN2-2 group image was accidentally used in Fig S1D HS578t si-NSUN2-2 group image.
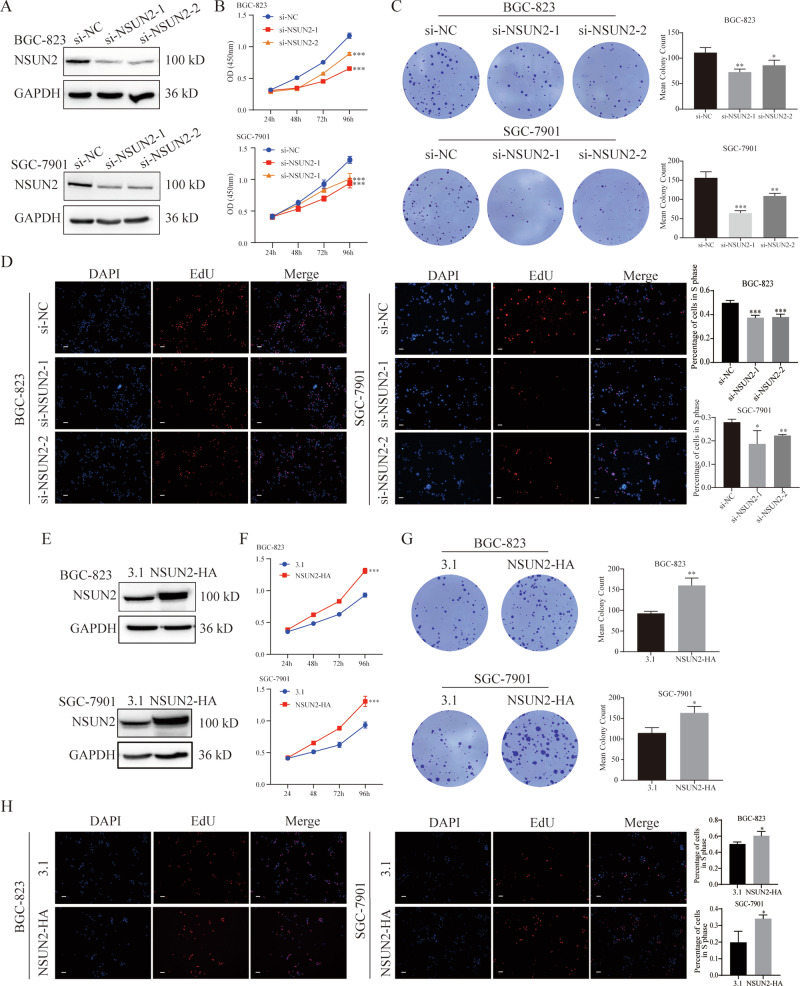

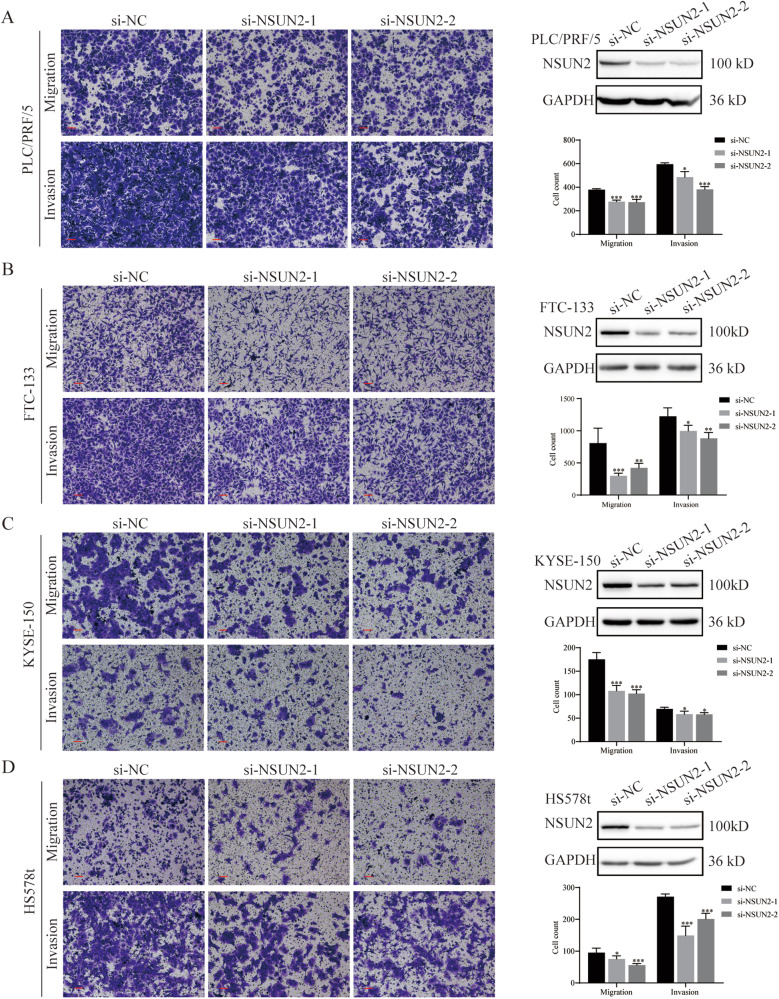


**Table 2 Univariate and multivariate Cox regression analysis of overall survival in patients with gastric cancer**.

3. Table 2. The age is (≥65 vs. <65); the differentiation is (poor vs. moderate + well).

**Table:** 

Variables	Univariate Cox analysis		Multivariate Cox analysis	
	HR (95% CI)	*P* value	HR (95% CI)	*P-*value
Gender (male vs. female)	1.45 (0.95–2.20)	0.082	1.56 (1.02–2.39)	0.042*
Age (≥65 vs. <65)	1.86 (1.33–2.61)	<0.001*	1.84 (1.31–2.58)	<0.001*
T (T3+T4 vs. Tis+T1+T2)	3.87 (2.39–6.27)	<0.001*	1.78 (0.95–3.34)	0.072
N (N2+N3 vs. N0+N1)	2.28 (1.64–3.16)	<0.001*	1.13 (0.74–1.74)	0.557
Stage (III+IV vs. I+II)	3.15 (2.19–4.55)	<0.001*	1.86 (1.16–2.97)	0.01*
Differentiation (poor vs. moderate + well)	2.10 (1.40–3.13)	<0.001*	1.71 (1.13–2.69)	0.01*
Tumor size (≥4.5 cm vs. <4.5 cm)	2.20 (1.59–3.05)	<0.001*	1.67 (1.19–2.34)	0.03*
NSUN2 expression (high vs. low)	1.69 (1.02–2.81)	0.042^*^	1.55 (0.93–2.59)	0.09

*Statistically significant (*P* < 0.05).

The original article has been corrected.

### Supplementary information

Supplementary Figure 1-corrected

