# Supplementary material for: NSUN2 modified by SUMO-2/3 promotes gastric cancer progression and regulates mRNA m5C methylation
Source: Cell Death Dis. 2021 Sep 9;12(9):842. doi: 10.1038/s41419-021-04127-3 (PMC8429414; doi:10.1038/s41419-021-04127-3)
Supplement: Supplementary file 1 — Supplementary Legends [file 41419_2021_4127_MOESM1_ESM.docx]

**Supplementary Figure 1. Transwell assays with four types of cancer cells after NSUN2 knockdown.** (A-D) Representative Transwell images showed that NSUN2 levels are closely related to the migration and invasion properties of hepatocellular carcinoma (PLC/PRF/5), thyroid cancer (FTC-133), esophageal cancer (KYSE-150), and breast cancer (Hs 578t). The knockdown efficiency of NSUN2 and the cell counts of the Transwell were showed on the right. Scale bar, 100 μm. *P < 0.05, **P < 0.01, and ***P < 0.001.

**Supplementary Figure 2. The effect of si-SUMO-2/3 on NSUN2 expression and localization.** (A) The silver staining of NSUN2 and its associated proteins. The red arrow represents overexpressed NSUN2. (B) The expression of NSUN2 was positively correlated with SUMO-2 and SUMO-3 in TCGA-STAD dataset. (C-D) The expression of NSUN2 and SUMO-2/3 on BGC-823 transfected with si-SUMO-2/3. (E) The cytoplasm and nuclear expression of NSUN2 on BGC-823 transfected with NSUN2-HA. (F) The cytoplasm and nuclear expression of NSUN2 on BGC-823 transfected with NSUN2-HA and si-SUMO-2/3. (G) Representative immunofluorescence images show that overexpressed NSUN2 significantly accumulates in the cytoplasm and that NSUN2 nuclear transport was blocked in NSUN2 knockout BGC-823 cells transfected with NSUN2-HA and si-SUMO-2/3. (H) Representative immunofluorescence images show that NSUN2-Δ236-240aa was expressed in the cytoplasm, whereas NSUN2-WT and NSUN2-Δ497-711 were mainly expressed within the nucleus, with some expression detected in the cytoplasm. Scale bar, 20 μm. (I) Transwell migration assay showing that SUMO-2/3 knockdown partially inhibits the tumorigenic effect of NSUN2 overexpression in NSUN2-KO cells. Scale bar, 100 μm. *P < 0.05, **P < 0.01, and ***P < 0.001.

**Supplementary Figure 3.** **NSUN2 promotes gastric progression via both m5C-dependent and -independent mechanisms.** (A) The number of migrating and invading cells in NSUN2-knockout BGC-823 transfected with NSUN2 wild type and mutant plasmids. (B) Network of enriched GO terms and bar graph of enriched KEGG terms across the unique m5C genes in the WT group. *P < 0.05, **P < 0.01, and ***P < 0.001. (C) Kaplan-Meier OS analysis for SHOC2, PCYT1A, PIK3R1, MT-ND4, and SREK1 in TCGA-STAD dataset. (D) The PIK3R1 and PCYT1A mRNA levels in WT and NSUN2 knockout BGC-823 cells, as determined via qRT-PCR. *P < 0.05, **P < 0.01, and ***P < 0.001.

**Supplementary Table 1. Sequences of siRNA.**

**Supplementary Table 2. Sequences of primers used for qRT-PCR.**
