# Supplementary material for: NSUN2 modified by SUMO-2/3 promotes gastric cancer progression and regulates mRNA m5C methylation
Source: Cell Death Dis. 2021 Sep 9;12(9):842. doi: 10.1038/s41419-021-04127-3 (PMC8429414; doi:10.1038/s41419-021-04127-3)
Supplement: Supplementary file 2 — Supplementary Table 1 [file 41419_2021_4127_MOESM2_ESM.docx]

**Supplementary Table 1 Sequence of siRNA**

| **Name** | **Sense (5’-3’)** |
| --- | --- |
| si-NSUN2-1 | GAAGCATCGTGCTGAAGTA |
| si-NSUN2-2 | GGGTTATCCTCACAAATGA |
| si-SUMO-2-1 (3’-UTR) | TGATACTGATGCCAAACAA |
| si-SUMO-2-2 (3’-UTR) | GGTCACTACAGTCTTTATT |
| si-SUMO-3-1 | CAGAGAATGACCACATCAA |
| si-SUMO-3-2 | GGCAGATCAGATTCAGGTT |
