# Supplementary material for: NSUN2 modified by SUMO-2/3 promotes gastric cancer progression and regulates mRNA m5C methylation
Source: Cell Death Dis. 2021 Sep 9;12(9):842. doi: 10.1038/s41419-021-04127-3 (PMC8429414; doi:10.1038/s41419-021-04127-3)
Supplement: Supplementary file 3 — Supplementary Table 2 [file 41419_2021_4127_MOESM3_ESM.docx]

**Supplementary Table 2 The primer sequences for RT-qPCR.**

| **Gene (Homo)** | **Sequence (5’-3’)** | |
| --- | --- | --- |
| SUMO-2 | Sense | ATGGCCGACGAAAAGCCCAAGGAAG |
|  | Anti-sense | CTTCATCCTCCATTTCCA |
| SUMO-3 | Sense | GAATGACCACATCAACCTGAAGG |
|  | Anti-sense | GCCCGTCGAACCTGAATCT |
| PIK3R1 | Sense | TGGACGGCGAAGTAAAGCATT |
|  | Anti-sense | AGTGTGACATTGAGGGAGTCG |
| PCYT1A | Sense | TCACGGTGATGAACGAGAATG |
|  | Anti-sense | CCTCTGTGTTGGAGCAAACAT |
| GAPDH | Sense | CAGGGCTGCTTTTAACTCTGGTAA |
|  | Anti-sense | GGGTGGAATCATATTGGAACATGT |
