# Supplementary figures and images for: NSUN2 modified by SUMO-2/3 promotes gastric cancer progression and regulates mRNA m5C methylation
Source: Cell Death Dis. 2021 Sep 9;12(9):842. doi: 10.1038/s41419-021-04127-3 (PMC8429414; doi:10.1038/s41419-021-04127-3)

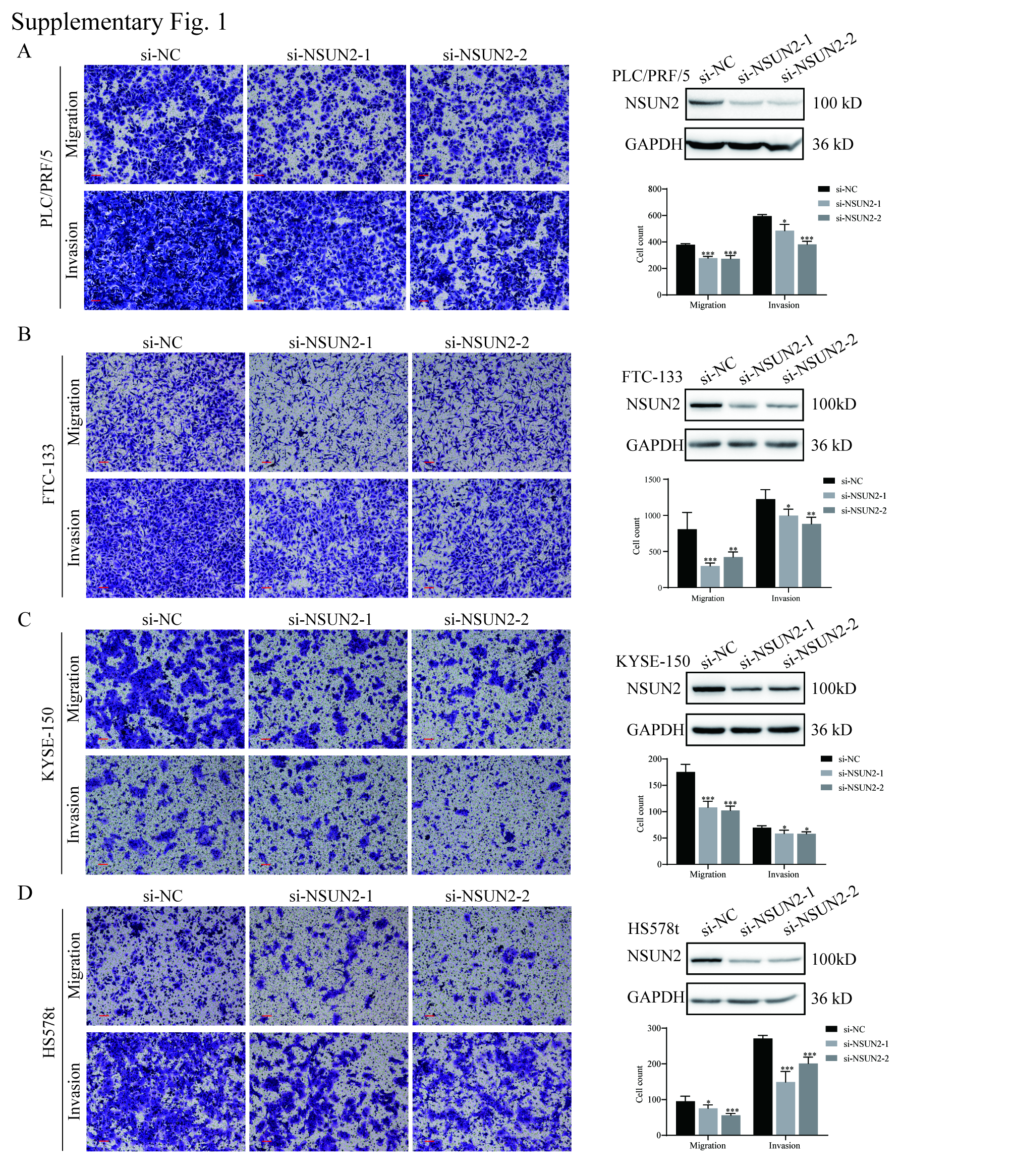

Supplement: Supplementary file 4 — Supplementary Figure 1 [file 41419_2021_4127_MOESM4_ESM.tif]

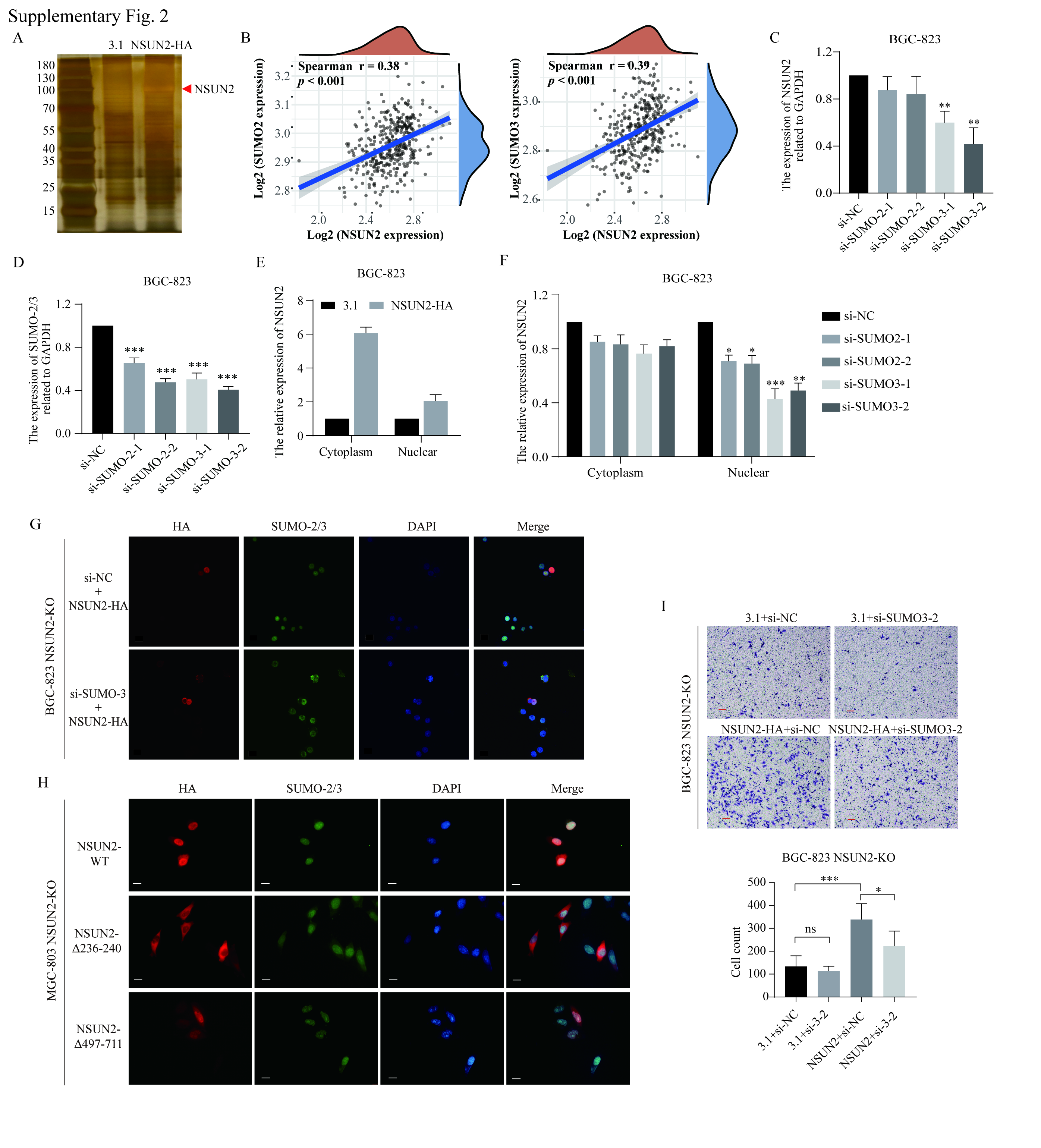

Supplement: Supplementary file 5 — Supplementary Figure 2 [file 41419_2021_4127_MOESM5_ESM.tif]

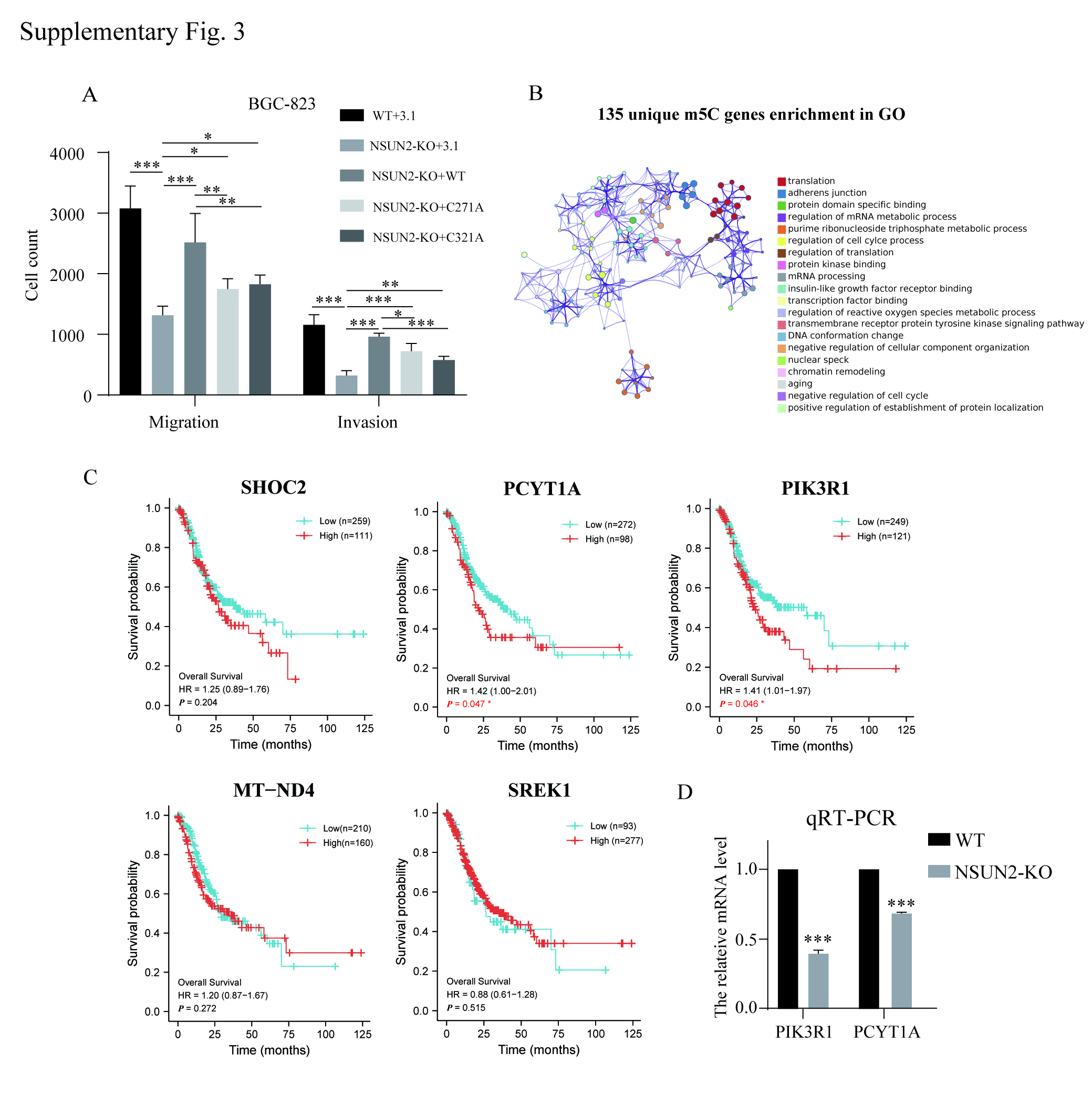

Supplement: Supplementary file 6 — Supplementary Figure 3 [file 41419_2021_4127_MOESM6_ESM.tif]
