# Supplementary figures and images for: Correction: NSUN2 modified by SUMO-2/3 promotes gastric cancer progression and regulates mRNA m5C methylation
Source: Cell Death Dis. 2024 Jul 11;15(7):495. doi: 10.1038/s41419-024-06859-4 (PMC11239835; doi:10.1038/s41419-024-06859-4)

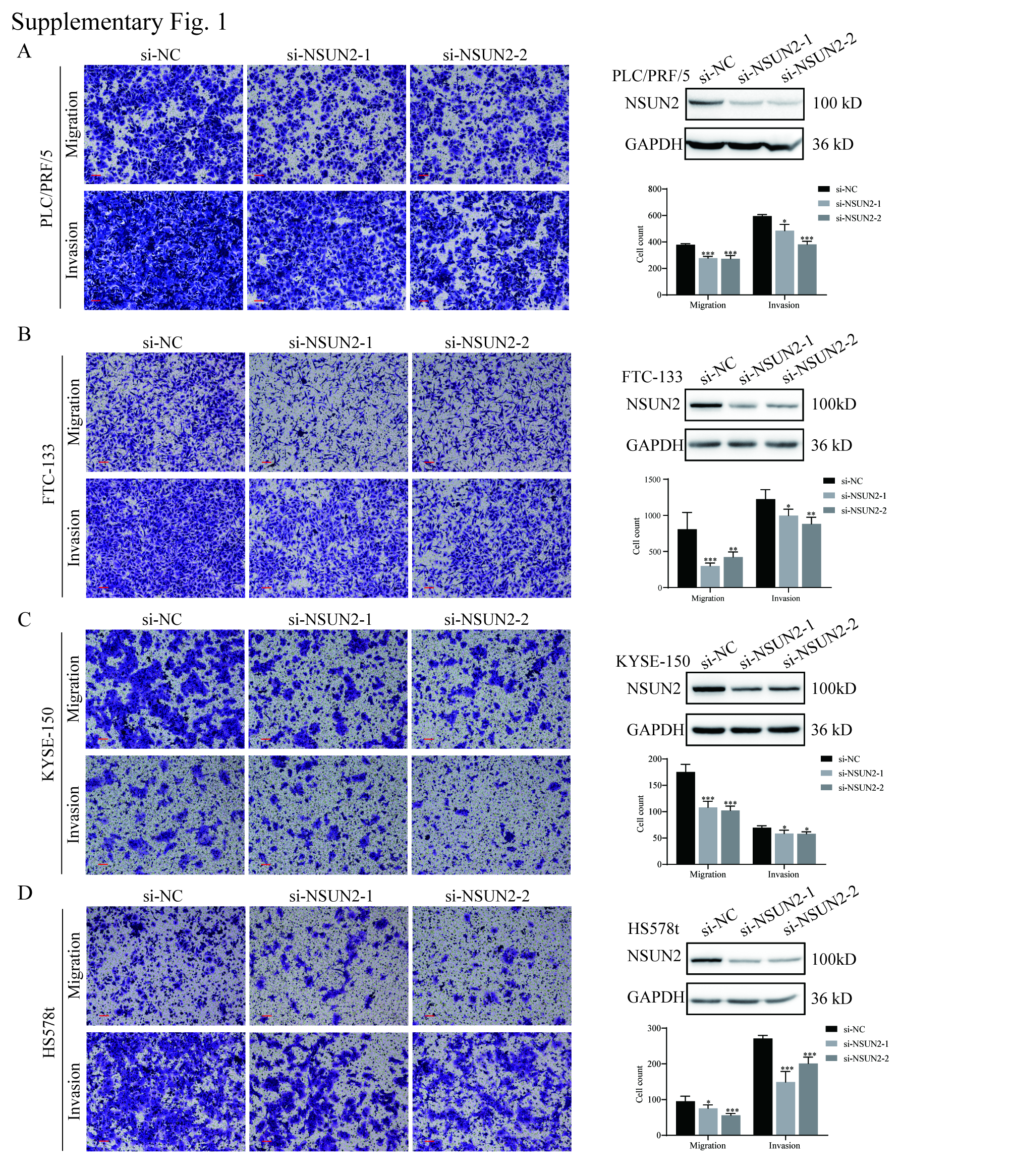

Supplement: Supplementary file 1 — Supplementary Figure 1-corrected [file 41419_2024_6859_MOESM1_ESM.tif]
